# Serum Dried Samples to Detect Dengue Antibodies: A Field Study

**DOI:** 10.1155/2017/7215259

**Published:** 2017-05-29

**Authors:** Angelica Maldonado-Rodríguez, Othon Rojas-Montes, Guillermo Vazquez-Rosales, Adolfo Chavez-Negrete, Magdalena Rojas-Uribe, Araceli Posadas-Mondragon, Leopoldo Aguilar-Faisal, Ana Maria Cevallos, Beatriz Xoconostle-Cazares, Rosalia Lira

**Affiliations:** ^1^Unidad de Investigacion Medica en Enfermedades Infecciosas y Parasitarias, UMAE Hospital de Pediatria, Centro Medico Nacional “Siglo XXI”, Instituto Mexicano del Seguro Social (IMSS), Mexico City, Mexico; ^2^Educacion e Investigacion en Salud, UMAE Hospital de Especialidades, Centro Medico Nacional “Siglo XXI”, Instituto Mexicano del Seguro Social (IMSS), Mexico City, Mexico; ^3^Medicina de Conservacion, Escuela Superior de Medicina, Instituto Politecnico Nacional, Mexico City, Mexico; ^4^Departamento de Biologia Molecular y Biotecnologia, Instituto de Investigaciones Biomedicas, Universidad Nacional Autonoma de Mexico, Mexico City, Mexico; ^5^Departamento de Biotecnologia y Bioingenieria, Centro de Investigacion y de Estudios Avanzados del Instituto Politecnico Nacional, Mexico City, Mexico

## Abstract

**Background:**

Dried blood and serum samples are useful resources for detecting antiviral antibodies. The conditions for elution of the sample need to be optimized for each disease. Dengue is a widespread disease in Mexico which requires continuous surveillance. In this study, we standardized and validated a protocol for the specific detection of dengue antibodies from dried serum spots (DSSs).

**Methods:**

Paired serum and DSS samples from 66 suspected cases of dengue were collected in a clinic in Veracruz, Mexico. Samples were sent to our laboratory, where the conditions for optimal elution of DSSs were established. The presence of anti-dengue antibodies was determined in the paired samples.

**Results:**

DSS elution conditions were standardized as follows: 1 h at 4°C in 200 *µ*l of DNase-, RNase-, and protease-free PBS (1x). The optimal volume of DSS eluate to be used in the IgG assay was 40 *µ*l. Sensitivity of 94%, specificity of 93.3%, and kappa concordance of 0.87 were obtained when comparing the antidengue reactivity between DSSs and serum samples.

**Conclusion:**

DSS samples are useful for detecting anti-dengue IgG antibodies in the field.

## 1. Introduction

Filter paper has been considered a good alternative for the collection, shipment, and storage of clinical samples, particularly in rural areas and low-resource settings. Several studies have been performed to address the utility of dried blood samples (DBSs) in the diagnosis of viral infections [1]. The demonstration of the stability of the samples in the dried format is one of the main challenges in these studies. A wide variety of target molecules, including antigens, antibodies, and nucleic acids, have been evaluated [1–4]. DBSs have been successfully used for the diagnosis of viral diseases, including HIV, HBV, HAV, chikungunya, and dengue [5–10].

In México, Dengue is the most prevalent arthropod-borne viral disease and is a severe public health problem [11]. The public health service cost is very high, and the recent emergence of other arboviruses has complicated the diagnosis due to the similarity of their clinical presentations [12]. The design of effective control measures is frequently complicated in low-resource settings because of the lack of infrastructure to perform the assays, and therefore the clinical samples need to be referred to specialized laboratories. Dried serum samples (DSSs) are easy to obtain in the field and can be shipped at room temperature (RT), which significantly reduces costs. Therefore, in low-income settings, epidemiological studies based on DSS would be ideal. The goal of this study was to evaluate the utility of DSSs for anti-dengue virus (DENV) antibodies in the field.

## 2. Materials and Methods

### 2.1. Patients

This cross-sectional study was conducted in Veracruz, Mexico, during the 2015 dengue outbreak. Blood specimens were collected by physicians in Hospital 71 of Veracruz, Mexico, from 66 patients with clinical suspicion of acute dengue virus infection. Patients presented, according to the WHO dengue case criteria, with a temperature > 38.5°C, myalgia, and exanthema. The study protocol was approved by the Ethics Committee and the Institutional Review Boards of the IMSS. Written informed consent was obtained from each participant.

### 2.2. Specimens

Blood was obtained from the vein. The sample was centrifuged to separate the serum, and two aliquots were prepared. One aliquot was stored and transported frozen at −20°C until use. The second aliquot was used to prepare the DSS samples. A Schleicher & Schuell #903 (S&S 903) filter paper card was prepared for each patient, and 30 *μ*l of serum was spotted onto each circle. Filter papers were dried overnight before transferring to a zip-lock plastic bag containing a silica gel desiccant and stored at RT until shipment to the virology laboratory in Mexico City (1 week after collection). Thereafter, filter paper cards were frozen at −20°C upon arrival to the laboratory. The paired serum and DSS samples were used within 6 months.

### 2.3. Elution of the Sample

The conditions for the optimal recovery of the samples from the filter paper were standardized. As an initial approach, absorbance of 280 nm was used as a proxy for the amount of protein eluted from the DSS. Different elution variables were tested, including temperature, time, and volume. The final elution conditions used in this study were as follows: for each test, a single spot per patient was used. The spot was cut into small pieces and placed into a 1.5 ml microcentrifuge tube containing 200 *µ*l of DNase-, RNase-, and protease-free PBS 1x (Fisher BioReagents, BP-2438-4). The sample was incubated for 1 h at 4°C, with shaking at 1,000–1,200 rpm in an orbital shaker. The supernatant was transferred to a clean 1.5 ml tube and kept at −20°C until use.

### 2.4. Dengue IgM and IgG Test

To detect anti-dengue antibodies, the DRG® Dengue Virus IgG kit (DRG International, Inc., USA) and the Anti-Dengue Virus ELISA (IgM) kit from EUROIMMUN (Lubeck, Germany) were used. Both assays were performed following manufacturer's recommendations. For serum samples, the recommended dilutions were used. For DSS samples, we determined the best volume of the eluted sample (10, 20, and 40 *µ*l) needed for the test using twelve of the serum samples already tested for anti-DENV antibodies and selected because they displayed a wide range against the virus (nine positive and three negative). The reactivity of samples that fell within the indeterminate range of the assay was defined as negative according to the instructions of the manufacturer.

### 2.5. Statistical Analyses

Data analyses were performed with the SPP software and EpiData 4.2 statistical program. Sensitivity, specificity, positive predictive value (PPV), and negative predictive value (NPV) were calculated by comparing the data of the assay performed using DSS samples with the results of the assay performed using the corresponding serum samples. Kappa concordance between paired DSS and serum samples was determined.

## 3. Results

### 3.1. Patients

Samples from 66 patients with suspected DENV infection were tested (Table 1). The samples were collected during the acute phase, approximately 4 days after the onset of fever. The average age was 34.6 ± 14.6 years.

### 3.2. DSSs Preparation

After separation of the serum, 30 *μ*l of sample was spotted into each circle of the filter card. We found that the 4 h drying time used in several studies was insufficient for the sample to dry in the highly humid city of Veracruz. Therefore, it was essential to let the samples dry at least overnight. Samples were kept at room temperature until being sent to Mexico City. Upon arrival at the laboratory, each sample was visually inspected to ensure complete dryness. Some samples, as required, were allowed to dry slightly longer.

### 3.3. Standardization of Conditions for the Elution

**Figure 1:**
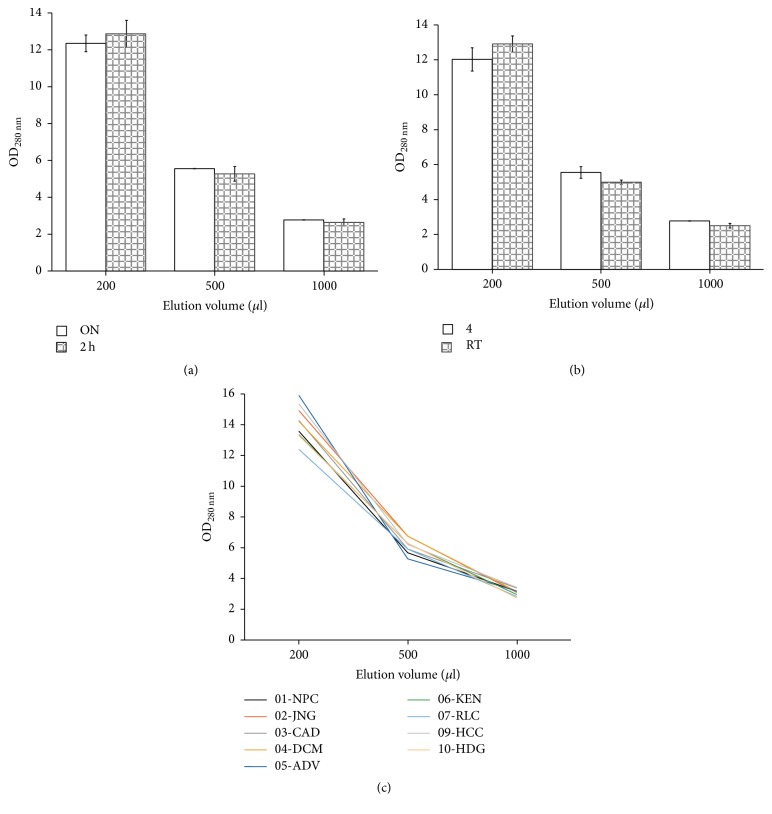
Standardization of sample elution. (a) Absorbance of eluates at 280 nm in samples incubated either at 2 h (empty bars) or overnight (ON, shaded bars) using different eluate volumes. (b) Absorbance of eluates incubated at either 4°C or room temperature (RT). (c) Absorbance of ten independent control samples eluates at different elution volumes.

We compared the amount of protein that could be eluted from the filter paper using different DNase-, RNase-, and protease-free PBS volumes, temperatures, and times (Figure 1). Independent of the conditions used, two hundred microliters was sufficient to elute the proteins present in the sample. The use of larger volumes only resulted in the proportional dilution of the sample. There were no differences when the samples were eluted either overnight or by only 2 h (Figure 1(a)). Similarly, there were no differences in the elution if it was carried out at RT or 4°C (Figure 1(b)). As preliminary results suggested that there was no difference in eluting samples for 1 or 2 h (data not shown), we decided to determine the amount of proteins eluted from ten DSSs containing normal serum at 4°C for only 1 h. The absorbance of the eluates at 280 nm ranged from 12.4 to 15.9, with elution at larger volumes only proportionally diluting the amount of protein obtained (Figure 1(c)). Therefore, our final elution conditions were 1 h at 4°C in 200 *µ*l of sterile DNase-, RNase-, and protease-free PBS 1x.

### 3.4. Standardization of the Sample Volume for Use in the IgG DENV ELISA Assay

**Figure 2:**
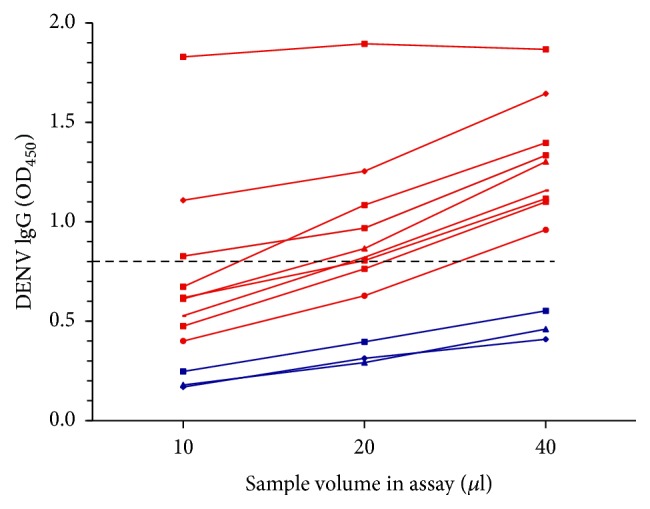
Standardization of the sample volume for use in the IgG DENV ELISA assay. Nine serum positive samples (red) and three negative samples (blue) were tested. Dashed line represents cut-off value.

As the ELISA kits are designed for use with liquid serum and not for dried samples, it was necessary to determine the amount of sample that gave the same results as the serum samples. The commercial kit used to detect anti-DENV IgG antibodies recommends diluting 10 *µ*l of serum into 1000 *µ*l of its proprietary sample diluent. The reactivity of the test sample is considered positive or negative depending on the 450 nm absorbance of the control standards (positive, negative, and cut-off). We evaluated three eluate volumes (10, 20, and 40 *µ*l) of known anti-DENV IgG positive and negative sera (Figure 2). When 40 *µ*l of the eluate was used, all positive samples gave a positive result, and the negative samples gave a negative result. The use of 10 or 20 *µ*l resulted in some false negative results.

### 3.5. Validation of the Dengue IgG Assay for DSS Samples

To validate the selected conditions, we tested the 66 serum and DSS paired samples obtained in the field. Results were analyzed in two ways: first by direct comparison of the level of absorbance obtained in the DENV-IgG tests (serum versus DSS) and second by comparing the level of agreement in the result of the test (either positive or negative). The correlation between the level of absorbance from eluates and serum samples was 0.79, which suggests that filter paper results reflect those found in serum over a wide range of IgG DENV reactivity.

**Figure 3:**
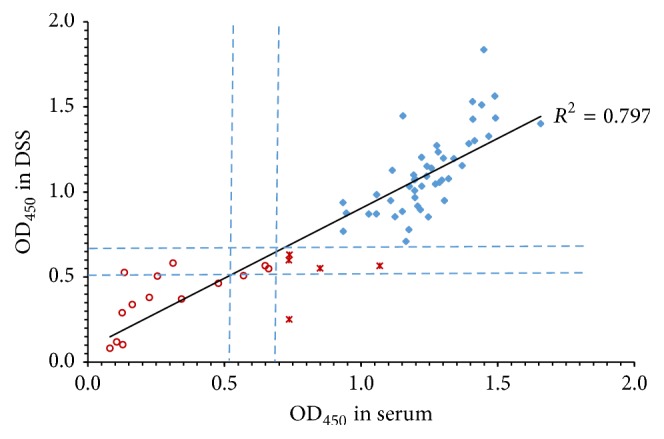
Correlation plot within anti-DENV-IgG reactivity in 66 paired DSS and serum samples. Red circles (○) represent negative samples in both tests, red asterisks (**∗**) represent samples that were positive in serum but negative in DSS, and blue rhomboids (⧫) represent positive samples in both tests. The cut-off values of the assays which define the gray zone are marked with dashed lines.

Comparison of the end results demonstrated that all negative sera (*n* = 14) were negative in the corresponding DSS sample and that the majority of the positives were positive (*n* = 47 of 52 samples), which indicates a close agreement between both collection systems, with kappa concordance of 0.87. Five of the DSS samples failed in IgG detection. Four of these five false negative results had low levels of reactivity in the serum assay (Figure 3). The fifth sample was clearly positive in the serum but it was within the gray zone in the DSS. The calculated sensitivity of the DSS assay was 94.2% and the specificity was 93.3%, with a positive predictive value (PPV) of 98% and a negative predictive value (NPV) of 87.5%.

It has been suggested that the presence of anti-IgM-DENV antibodies correlates with acute infection. Therefore, we investigated the utility of DSS samples for the detection of IgM antibodies. There were only four samples that were positive for IgM in the serum assay, and only one of these was also positive in the DSS assay. Among the 62 serum IgM negative samples, there were no false positive results. Due to the small positive sample size, no further analysis was performed.

## 4. Discussion

Dengue is an important public health problem in Mexico. It has been reported that Veracruz has the largest number of dengue and dengue hemorrhagic fever cases in Mexico [13]. Therefore, it is important to develop inexpensive and efficient sampling tools for continuous epidemiological surveillance.

During dengue disease outbreaks, the shipping of samples to specialized laboratories is important to monitor the magnitude of the outbreak. Transportation of these samples from austere field environments to reference laboratories requires the maintenance of a cold chain during transport which might not be available or could be prohibitively expensive. The use of alternative methods of sample collection, such as the DBS or DSS samples, might overcome these problems. These methods are cheap and easy to perform in field environments, requiring minimal sophisticated equipment and personal training [9].

In this study, we evaluated the utility of the DSS samples prepared in the field to detect anti-DENV antibodies. Because one of the most important processes in the laboratory diagnosis is the quality of the sample, the process of sample collection and elution is critical. First, it is imperative that the sample is completely dry before transferring to plastic bags. The recommended drying time for DBS is at least 4 h or overnight [14]. In our study, performed in a highly humid place, complete dryness took at least overnight. In Cuba, Herrera et al. [15] also had to let their samples dry overnight before storage. In our experience, when the DSS samples are not completely dried, antibodies can become degraded. The time and conditions for storage of DSSs before processing have not been systematized. Depending on the study, temperature of the storage of the filter paper has been done at RT, 4°C, or −20°C [15–18]. Documented time of filter paper storage also varied from weeks to a year [10, 15, 17, 19]. In general, for diagnosis of other infectious diseases, it has been recommended that the samples collected on filter paper should only be stored at RT for a maximum of 2 weeks and that the prolonged storage should be at −20°C [1].

The methods for the elution of the samples have also been largely empirical and therefore need to be standardized to maximize the performance of specific tests. Previous studies using filter paper samples to detect anti-DENV antibodies have used a variety of elution buffers, including PBS alone, PBS with Tween, fetal calf serum, or albumin [10, 18–21]. There is no consensus regarding the volumes, times, and temperatures to elute the samples. We decided to study the use of PBS only because it has been successfully used to elute dried samples in the detection of a wide range of molecular markers including DENV, HBV, and herpes simplex virus [16, 22, 23]. We reasoned that if samples could be eluted in the absence of other additives such as protein, serum, or detergents, it would be easy to use part of the elution in other assays such as determination of viral loads without fear of interference from these additives. We compared different elution conditions and found that elution with 200 *µ*l of sterile protease- and nuclease-free PBS for 1 h at 4°C gave reproducible results. The amount of eluate used in the assay was also standardized to give similar results to those found in serum.

Using our protocol, we achieved a good agreement between the filter paper and serum results for IgG antibody detection, similar to those obtained in other studies [10, 18]. It was not possible to evaluate the value of the test for detection of IgM antibodies, as only four serum samples were positive. It is worthwhile to note that only one of the four samples was positive in the DSS test and that we did not identify any false positive test. It has been reported that anti-DENV IgM test is maybe less stable than IgG antibodies on filter paper and this may contribute to the low positive rate identified in this study [9, 10]. A second reason may be the fact that the outbreak study was due to the presence of both DENV and chikungunya (CHIKV) in the area. The presence of CHIKV in Mexico was reported during the time when these samples were collected [24]. However, the objective of the present study was to evaluate the use of DSS in the diagnosis on DENV and not the specific cause of fever in these patients.

We have demonstrated the feasibility of using filter paper as a collection method for the detection of IgG antibodies in the field. This is important because many countries, where dengue is endemic, lack infrastructure to appropriately store and ship serum samples. Furthermore, the use of filter paper not only is less expensive but also reduces the risk of spillage of infectious material [14]. We propose that DSS assays are useful in DENV epidemiological studies. The presence of anti-DENV IgM antibodies could be useful to establish the differential diagnosis with CHIKV. DSSs can also be applied to all emergent arboviruses that are cocirculating currently in our country, such as CHIKV and Zika (ZIKV), thus reducing costs of the epidemiological studies.

## 5. Conclusions

Although serum samples are considered the standard tests for serological detection of DENV antibodies, we have demonstrated that filter paper is a feasible method of sample collection in the field which gives equivalent results to those obtained with serum samples. The standardized protocol is easy to perform; the resulting samples are cheap to transport and stable for months and require less storage space.

More studies should be carried out to validate the use of the dried samples not only for diagnosis but also for epidemiology and surveillance in low-resource countries.

## Figures and Tables

**Table 1:** Clinical and demographic patient data.

Patients	*n* = 66
Sex (M : F)	37 : 29
Age (years)	34.6 ± 14.6
Onset fever (days) average	3.7
